# Selenium Accumulating Leafy Vegetables Are a Potential Source of Functional Foods

**DOI:** 10.1155/2015/549676

**Published:** 2015-12-10

**Authors:** Petro E. Mabeyo, Mkabwa L. K. Manoko, Amra Gruhonjic, Paul A. Fitzpatrick, Göran Landberg, Máté Erdélyi, Stephen S. Nyandoro

**Affiliations:** ^1^Chemistry Department, College of Natural and Applied Sciences, University of Dar es Salaam, P.O. Box 35061, Dar es Salaam, Tanzania; ^2^Botany Department, College of Natural and Applied Sciences, University of Dar es Salaam, P.O. Box 35060, Dar es Salaam, Tanzania; ^3^Sahlgrenska Cancer Centre, University of Gothenburg, 405 30 Gothenburg, Sweden; ^4^Department of Chemistry and Molecular Biology, University of Gothenburg, 412 96 Gothenburg, Sweden

## Abstract

Selenium deficiency in humans has been associated with various diseases, the risks of which can be reduced through dietary supplementation. Selenium accumulating plants may provide a beneficial nutrient for avoiding such illnesses. Thus, leafy vegetables such as* Amaranthus hybridus*,* Amaranthus *sp.,* Cucurbita maxima*,* Ipomoea batatas*,* Solanum villosum*,* Solanum scabrum*, and* Vigna unguiculata* were explored for their capabilities to accumulate selenium when grown on selenium enriched soil and for use as a potential source of selenium enriched functional foods. Their selenium contents were determined by spectrophotometry using the complex of 3,3′-diaminobenzidine hydrochloride (DABH) as a chromogen. The mean concentrations in the leaves were found to range from 7.90 ± 0.40 to 1.95 ± 0.12 *μ*g/g dry weight (DW), with* C. maxima* accumulating the most selenium. In stems, the accumulated selenium content ranged from 1.12 ± 0.10 *μ*g/g in* Amaranthus* sp. to 5.35 ± 0.78 *μ*g/g DW in* C. maxima* and was hence significantly different (*P* < 0.01). The cancer cell line MDA-MB-231 was used in cytotoxicity assays to determine the anticancer potential of these extracts. With exception of* S. scabrum* and* S. villosum*, no cytotoxicity was detected for the selenium enriched vegetable extracts up to 100 *μ*g/mL concentration. Hence, following careful evaluation the studied vegetables may be considered as selenium enriched functional foods.

## 1. Introduction

Selenium is a semimetallic, biologically active trace element that forms bonds with carbon whose strength is in the range of common covalent bonds. Commonly occurring in selenoamino acids and selenoproteins [1, 2], selenium is involved in the regulation of a variety of cellular functions, including enzyme catalysis and signal transduction. It also plays an essential role as a trace element necessary for brain function [3]. In its organic forms selenium may act as an antioxidant, preventing DNA damage, and may also counteract heavy metal toxicity, thereby preventing cancer and further degenerative diseases [4–8]. Still, it may also inhibit cell growth and nucleic acid protein synthesis and induce cell death [9, 10]. Selenium deficiency affects glutathione metabolism (GSH) and glutathione peroxidase (GSHPx) activity and may thereby facilitate the development of cell-mediated-intracellular infections, such as tuberculosis. Low serum selenium is seen as a risk factor for mycobacterial infection for human immunodeficiency virus positive individuals [11]. Organoselenium compounds are chemoprotective against a number of human and animal viral infections [12, 13]. They also promote formation of sperms. A low dietary selenium intake is associated with oxidative stress-related conditions, reduced fertility, immune dysfunctions, increased cancer risk [12], senility, and the development of Keshan, Kashin-Beck, and Alzheimer's diseases [9, 14]. Dietary selenium intake, by simple means such as via vegetables capable of accumulating it, is therefore of vital importance.

Whereas selenium is essential as trace element, it can cause acute or chronic toxicity at higher serum concentrations [15–17]. Its effect at elevated concentrations is associated with memory loss, fatigue, musculoskeletal complaints, nausea, nail discoloration or brittleness, muscle pain and cramps, joint pain, and gastrointestinal disorders, such as diarrhoea and vomiting [17]. Out of all elements, selenium has the narrowest range between dietary deficiency (less than 40 *μ*g/day) and toxicity (greater than 400 *μ*g/day) [15], requiring careful control of its human and animal intake. Naturally, the selenium dietary intake shows a geographical variation, which is influenced by the selenium content of soils on which the food is grown and by the local eating habits [18, 19]. The requirement for selenium intake is commonly estimated based on the concentration needed to maximize the activity of plasma selenoproteins and of glutathione peroxidase, the latter being an oxidant defence enzyme [19]. Although there is no universal dietary reference for selenium, a variety of studies were performed to establish the reference intake that reduces the risk of selenium related human diseases [20]. These resulted in a variety of selenium dietary intakes depending on country, organization, and sex and age groups. To date, a 55 *μ*g Se/day intake is commonly accepted as the reference labelling value (RLV) [19, 21].

**Figure 1:**
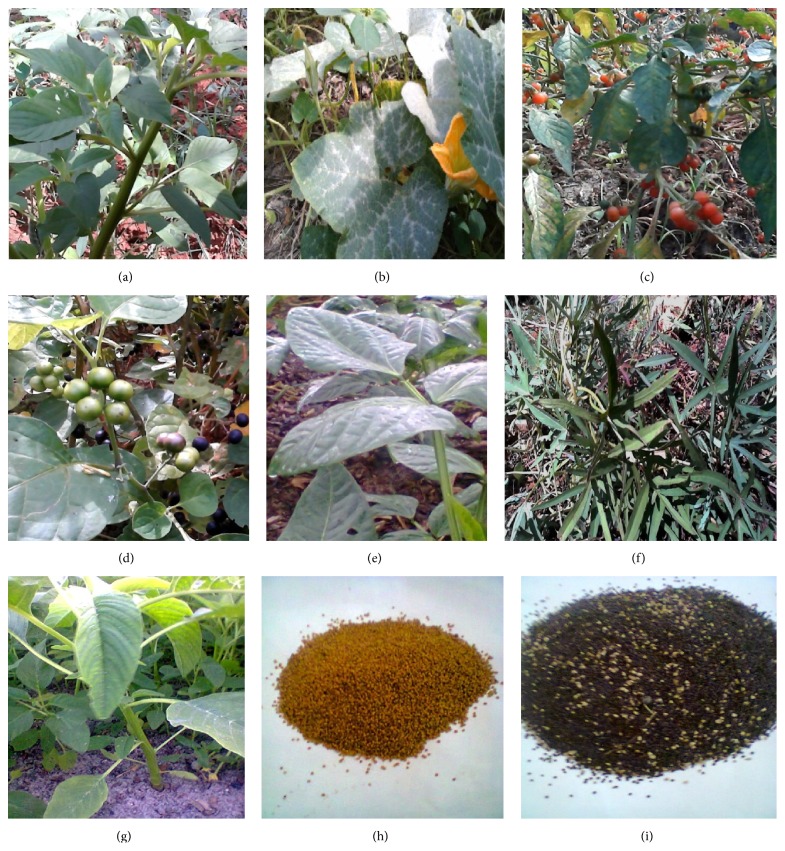
(a)* Amaranthus hybridus*, (b)* Cucurbita maxima*, (c)* Solanum villosum*, (d)* Solanum scabrum*, (e)* Vigna unguiculata*, (f)* Ipomoea batatas*, (g)* Amaranthus* sp., (h)* Amaranthus hybridus* seeds, and (i)* Amaranthus* sp. seeds.

Humans and animals commonly obtain selenium from cereals, grains, and vegetables grown on seleniferous soils and from animal products such as meat, milk, fish, and eggs [22–25]. Plants so far studied for their ability to accumulate selenium include* Allium sativum* (garlic) [26],* A. cepa* (onions) [27],* A. fistulosum* (green onion) [28],* Brassica juncea* (mustard) [29, 30],* Brassica oleracea* (wild cabbage and related cultivars) [31],* Astragalus bisulcatus* (milk-vetch) [32, 33],* A*.* racemosus* (cream or alkali milk-vetch) [33],* Cucurbita pepo* (summer squash) [34, 35], and* Stanleya pinnata* (desert princesplume) [29, 30]. In this study, the yet unexplored leafy vegetables* Amaranthus hybridus* (smooth amaranth, smooth pigweed, and red amaranth),* Amaranthus* sp. (unidentified),* Cucurbita maxima* (pumpkin),* Ipomoea batatas* (sweet potato, a nontuberous variety),* Solanum scabrum* (African nightshade, black nightshade),* S. villosum* (woolly nightshade, red-fruit nightshade), and* Vigna unguiculata* (cowpeas) (Figure 1) were investigated for their selenium accumulating capabilities in order to explore their possible use as functional foods. Despite the scanty consumption records available for these investigated vegetables, they are commonly grown and consumed by various communities in Eastern Africa, a region naturally deprived of soil selenium content. The plants were grown on selenium enriched soil and their selenium content was determined by spectrophotometric detection of the chromogen selenium-3,3′-diaminobenzidine hydrochloride (DABH) complex. The cytotoxicity of the vegetables extracts was assessed against the MDA-MB-231 human breast cancer cell line.

## 2. Experimental Section

### 2.1. Vegetable Cultivation

The selected leafy vegetable species were grown on selenium enriched soil (except for control purpose) at the nursery site of the Botany Department, University of Dar es Salaam, following standard procedures [36]. To enrich the soil, 20 L of 0.65 mg/L Na_2_SeO_4_ solution was applied to each 100 m^2^ portion of the nursery. During the growth period, watering during the morning and evening every day and regular weeding were carried out.

### 2.2. Sample Collection and Preparations

Vegetable samples were harvested at early and late stages of their growth. Early sampling was done at the age of three weeks after seed germination for* Amaranthus* spp.,* V. unguiculata*, and* C. maxima* while* S. villosum*,* S. scabrum*, and* I. batatas* were harvested at the age of six weeks based on differences in growth rate. The late harvest was done three weeks after the early harvest of each vegetable species. Samples were rinsed with tap water, followed by distilled water, and were then separated into stems and leaves. The samples were chopped into pieces, packed in separate plastic bags and transferred to the laboratory for oven drying to constant weight at a temperature between 50°C and 60°C, and ground to powder following an established procedure [37]. For* Amaranthus* spp., seeds were also harvested. The seeds were first left to mature and dry and then harvested and further air-dried before being ground to powder for homogenization. Powdered seeds, leaves, and stem samples were stored in a freezer (at −4°C) prior to analysis.

Soil samples were collected in three stages that involved random sampling using shovel. The first collection was done immediately after nursery preparation before sowing seeds and application of selenium. This was necessary in order to determine any remaining traces of selenium left in the soil as 10 g Se/ha was applied to the area three years earlier before this study. Following application of selenium, on the next day, random sampling to collect a second set of soil samples was done. The soil samples were then air-dried, packed in plastic bags, and stored in the freezer (at −4°C) until the analysis. After all vegetable samples and seeds were harvested; the third set of soil sample was collected to determine the amount of selenium left in the soil.

### 2.3. Chemicals and Reagents

Analytical grade reagents and HPLC grade solvents were used as received from commercial suppliers, without further purification: nitric acid (HNO_3_; 70%), hydrogen peroxide (H_2_O_2_; 30%), toluene, concentrated ammonia (NH_3_) solution, 0.5 mol L^−1^ sulphuric acid, and 3,3′-diaminobenzidine hydrochloride (DABH) solution that was freshly prepared by dissolving 0.125 g 3,3′-DABH in 25 mL acetone and then stored in amber-coloured bottle. A solution of sodium selenate (Na_2_SeO_4_) was used both for soil application and as a standard. Deionized water was used for preparing reagent solutions and samples throughout.

### 2.4. Equipment and Tools

To dry vegetable samples, a Genlab Ltd. oven was used. During sample digestions and reaction of 3,3′-DABH and selenium in the sample a microwave oven (Sonash Co.) was applied. A Shimadzu-240 UV spectrophotometer equipped with 1 cm high quartz and silica cuvettes was used for reading the absorbance of the Se-3,3′-DABH complex (piazselenol). All laboratory glassware was kept overnight in 10% nitric acid solution to remove contaminants and was rinsed with deionized water and dried in a dust-free oven ahead of use. Whatman cellulose filter papers were used for filtration of the digested samples in order to remove the remained silica.

### 2.5. Preparation of Standard Solution and Calibration Curve

For determination of the selenium content of extracts the method established by Katamto and Al-Zehouri was adopted [34]. A 920 *μ*g/mL stock solution was prepared by dissolving 0.219 g Na_2_SeO_4_ in 100 mL deionized water; appropriate amounts were diluted with deionized water (pH adjusted to 1.8 ± 0.1 using 0.5 M H_2_SO_4_) to 0.1 *μ*g/mL, 0.2 *μ*g/mL, 0.3 *μ*g/mL, 0.5 *μ*g/mL, 0.7 *μ*g/mL, 0.9 *μ*g/mL, and 1.1 *μ*g/mL concentrations, which were used for construction of a calibration curve. The absorbance of selenium plotted against concentration gave a linear correlation, which was then used to determine the unknown selenium concentration of the samples.

### 2.6. Sample Digestion and Total Se Concentration Determination with 3,3′-DABH

A 1 g portion of each of the powdered dry leaves, stems, seed, and soil sample was digested with 12 mL concentrated nitric acid (70% HNO_3_) and 4 mL hydrogen peroxide (30% H_2_O_2_) in 100 mL conical flasks. The mixture was subjected to heat at 70 ± 5°C for 35 minutes using a microwave oven. The solution was filtered and diluted to 100 mL with deionized water. The selenium concentration was determined by spectrophotometric analysis using 3,3′-diaminobenzidine hydrochloride (DABH) as chromogen [38]. Thus, aliquotes of a 10 mL sample solution were transferred into a series of 30 mL heat resistant vials, 0.5 mL 3,3′-DABH was added, and the mixture was heated to 70°C for 20 min. The mixture was then cooled and the pH adjusted to 8.0 ± 1.0 using concentrated ammonia solution (NH_3_). The colored complex was extracted with 10 mL toluene and its absorbance was measured at 420 nm. Standard deviation (SD) was calculated for triplicate measurements and the concentration was calculated using the regression equation of the concentration versus UV absorbance calibration (*vide supra*).

### 2.7. Anticancer Assay

Cytotoxic activity of the aqueous vegetable extracts was evaluated against the MDA-MB-231 human cancer cell line as previously reported by Irungu et al. [39]. MDA-MB-231 cells were cultured in Dulbecco's modified Eagle medium supplemented with 10% (v/v) fetal bovine serum, 2 mM L-glutamine, 100 units/mL penicillin, and 100 *μ*g/mL streptomycin at 37°C in humidified 5% CO_2_. Cells were seeded in 96-well plates at optimal cell density (10 000 cells per well) to ensure exponential growth for the duration of the assay. After a 24 h preincubation growth, the medium was replaced with experimental medium containing the appropriate extract concentrations (100–0.1 *μ*g/mL) or vehicle controls (0.1% or 1.0% v/v DMSO). After 72 h of incubation, cell viability was measured using Alamar Blue reagent (Invitrogen AB, Lidingö, Sweden) according to the manufacturer's instructions. Absorbance was measured at 570 nm with 600 nm as a reference wavelength. Results were expressed as mean ± standard error for six replicates as a percentage of vehicle control (taken as 100%). Extract that showed <80% viability was considered as a positive hit for cytotoxicity. Such extracts were further subjected to new cell library testing under serial dilutions to obtain LD_50_. Experiments were performed independently at least six times followed by statistical analyses.

### 2.8. Statistical Data Analysis

Comparison of the concentrations of Se accumulated in different leafy vegetable species was achieved using Kruskal-Wallis Analysis of Variance (ANOVA) and Dunn's Multiple Comparison test. Statistical analyses for cytotoxicity assay were performed using a two-tailed Student's *t*-test whereby *P* < 0.05 was considered to be statistically significant.

## 3. Results and Discussion

### 3.1. Selenium Uptake

The vegetables shown in Figure 1 were grown on selenium enriched soil, using Na_2_SeO_4_ solution (for details, see Section 2), at the Botany Department of the University of Dar es Salaam following a standard procedure [36]. As control, plants grown without selenium enrichment were used.

The selenium concentration of the vegetable extracts was determined by UV spectrophotometric analyses with detection of the chromogen 3,3′-diaminobenzidine hydrochloride- (DABH-) selenium complex at *λ* = 420 nm [38]. For validation, samples containing known concentrations of selenium were investigated, providing linear response with correlation coefficient (*R* ^2^) of 0.9895, as summarized in Table 1.

All seven investigated leafy vegetable species were found to accumulate selenium; that is, they possessed higher concentrations than the controls (Table 2), albeit to a varying extent. The highest selenium concentration was found in* C. maxima*, 7.90 ± 0.40 *μ*g/g and 6.49 ± 0.26 *μ*g/g dry weight (DW) for late and early harvest, respectively, while lowest concentration was found in* Amaranthus* sp., 2.21 ± 0.09 *μ*g/g and 1.95 ± 0.12 *μ*g/g DW, for late and early harvest, respectively.

The stems of some leafy vegetables, such as those of* Amaranthus* and* Solanum*, are usually consumed together with their leaves. Determination of the selenium content of the stems indicated selenium accumulation, with a similar trend in between the species to that observed for the leaves, yet in lesser extent (Table 2). Among the early harvested stem samples* Amaranthus* sp. accumulated the lowest amount of selenium (1.12 ± 0.10 *μ*g/g DW) whereas* V. unguiculata* the highest (3.39 ± 0.41 *μ*g/g DW); among the samples harvested at a late time point, the lowest concentration was observed for* Amaranthus* sp. (1.83 ± 0.05 *μ*g/g DW) and the highest for* C. maxima* (5.35 ± 0.78 *μ*g/g DW) (Table 2).

Variance analyses indicated that the increased selenium concentration of the vegetables that were grown on selenium enriched soil was statistically significant, when compared to the control samples. The differences between late and early harvests of leaves and stems were, however, not significant (Table 3). Thus, the time of harvesting within a three-to-six-week interval is of no importance for their use as functional foods. However, daily dietary intake has to be taken into consideration to ensure that the required amount for health benefits is taken, not to a toxic or deficient level.

Statistical analyses (Supporting Information in Supplementary Material available online at http://dx.doi.org/10.1155/2015/549676) indicated that the difference in selenium uptake for leaves was significant only in between* C. maxima* and* Amaranthus* sp. (*P* < 0.01) but not the other plants. For the stem samples, the difference in selenium uptake was statistically significant between* V. unguiculata* and* Amaranthus* sp. for early harvested samples (*P* < 0.05) and between* C. maxima* and* Amaranthus* sp. (*P* < 0.01) for late harvested stems. Although the differences of the selenium uptake of various plants were not statistically significant, they may still have a biological impact considering the amount of plants consumed by a human.

During the growth stage, selenium is usually found in the leaves whilst during the reproductive stage it is accumulated in the seeds [40, 41]. This is of importance, as the seeds of* Amaranthus* species are taken as food in the form of porridges or bread as cereal substitute by some communities. Therefore the selenium accumulation in the seeds of two* Amaranthus* spp. was also studied, the results being given in Table 4. Seeds of* A. hybridus* and* Amaranthus* sp. amassed an average selenium content of 2.27 ± 0.18 *μ*g/g and 1.26 ± 0.06 *μ*g/g DW, respectively, that is, concentrations that were lower than those found in the respective leaves, but either higher or lower in the respective stems. This may imply that vegetative parts accumulate more selenium than reproductive parts or that certain organs of plants accommodate some forms of selenium with higher preference.

Soils ability to retain minerals may allow a single selenium enrichment to provide soil for functional foods for more than a single season and is thus of economic importance for farmers. Selenium concentrations of the soil prior to and following the enrichment, that is, immediately after the harvest, were measured, the results being summarized in Table 5. Here it should be noted that traces of selenium were present in the soil before enrichment due to a previous study at the Botany Department of the University of Dar es Salaam three years earlier, which in fact explains the trace amounts of selenium found in the control samples.

The accumulated selenium concentrations of the investigated vegetable samples ranged from 1.12 ± 0.10 to 7.90 ± 0.40 *μ*g/g DW, whereas the selenium concentration of the soil following the harvest was 1.05 ± 0.26 *μ*g/g DW. Thus all seven investigated vegetables are accumulators. However, it should be noted here that some decrease of the soil selenium concentration may have been due to leaching and not due to bioaccumulation.

### 3.2. Anticancer Potential

To determine the anticancer potential of the selenium enriched vegetables, the aqueous extracts were studied for their ability to inhibit the growth of the human MDA-MB-231 cancer cell line (Table 6). With exception of* S. scabrum* and* S. villosum*, which exhibited cytotoxicity at 100 *μ*g/mL for both the control and selenium enriched leaves extracts, no cytotoxicity was detected for the rest of the samples. Hence, the marginal cytotoxicity of* Solanum* species is attributable to their phytochemical constituents, notably solanine, solasodine, or solanidine alkaloids [42, 43], and not to their selenium content. It should be noted that* Solanum* species may be administered at a dose range of 200 mg/kg body weight without any side effects, as food or for medical purposes [43]. To ensure safe application of the studied vegetables as functional foods for selenium intake, further* in vivo* and clinical toxicological assessments are needed.

## 4. Conclusions

Seven vegetable species were identified as selenium accumulators with their selenium content being within the WHO-determined safe, nontoxic concentration, <1400 *μ*g/L [15, 16]. The intake of approximately 10–25 g dry weight of these vegetables is required to meet the 55 *μ*g Se/day recommended dietary dose of selenium [19, 44]. They can be cultivated on selenium enriched soils to accumulate selenium and are therefore excellent candidates to be developed to functional foods.

## Supplementary Material

Statistical analyses (Table S1) indicated that the difference in selenium uptake of leaves was significant only in between C. maxima and Amaranthus sp (p<0.01), but not the other plants. For the stem samples, the difference in selenium uptake was statistically significant between V. unguiculata and Amaranthus sp for early harvested samples (p<0.05) and between C. maxima and Amaranthus sp (p<0.01) for late harvested stems.

## Figures and Tables

**Table 1:** Method validation: the concentration of extracts possessing known amounts of selenium determined by gravimetric analyses and by UV spectrophotometry.

Concentration [Se (VI)] spiked (*µ*g/mL)	Mean absorbance	Measured concentration (*µ*g/mL)	Recovery (%)	Recovery (%) ± SD
1	0.06	0.93	93.10	94.56 ± 1.44
3	0.17	2.88	95.97	
5	0.27	4.73	94.60	

**Table 2:** Selenium concentration (*µ*g/g) in vegetable samples.

Vegetable species	Mean concentration (*µ*g/g) ± SD					
	Leaves samples			Stems samples		
	Early harvest	Late harvest	Control	Early harvest	Late harvest	Control
*V. unguiculata*	5.86 ± 0.46	7.01 ± 0.21	0.75 ± 0.16	3.39 ± 0.41	4.37 ± 0.13	0.56 ± 0.09
*C. maxima*	6.49 ± 0.26	7.90 ± 0.40	1.30 ± 0.27	2.32 ± 0.32	5.35 ± 0.78	0.56 ± 0.13
*A. hybridus*	2.37 ± 0.37	2.81 ± 0.09	0.28 ± 0.06	1.22 ± 0.09	1.97 ± 0.02	0.19 ± 0.02
*Amaranthus *sp.	1.95 ± 0.12	2.21 ± 0.09	0.22 ± 0.07	1.12 ± 0.10	1.83 ± 0.05	0.16 ± 0.16
*S. villosum*	3.05 ± 0.69	4.48 ± 1.61	0.38 ± 0.02	2.39 ± 0.40	2.57 ± 0.60	0.37 ± 0.01
*S. scabrum*	3.25 ± 0.36	4.11 ± 0.99	0.33 ± 0.02	2.53 ± 0.05	2.73 ± 0.10	0.14 ± 0.02
*I. batatas*	4.13 ± 0.41	5.56 ± 1.42	0.43 ± 0.02	3.10 ± 0.21	3.08 ± 0.18	0.45 ± 0.04

**Table 3:** The one-way ANOVA for the late, the early, and the control harvests of vegetables.

Test samples	*P* value	Average	Variances	Differences
LLH versus CS	1.61675*E* − 4	4.86943	4.3871	*∗∗*
		0.52871	0.14622	

LSH versus CS	1.06056*E* − 4	3.12829	1.65798	*∗∗*
		0.34643	0.03463	

ELH versus CS	3.11278*E* − 4	3.871	2.98636	*∗∗*
		0.52871	0.14622	

ESH versus CS	7.71689*E* − 5	2.29643	0.74049	*∗∗*
		0.34643	0.03463	

LLH versus ELH	0.34985	4.86943	4.3871	ns
		3.871	2.98636	

LSH versus ESH	0.18075	3.12829	1.65798	ns
		2.29643	0.74049	

ELH: early leaf harvest, LLH: late leaf harvest, ESH: early stem harvest, LSH: late stem harvest, CS: control samples, *∗∗*: very significant difference, and ns: no significant difference.

**Table 4:** Selenium concentrations observed for *Amaranthus* seeds.

Vegetable species	Concentration *µ*g/g			Mean concentration, (*µ*g/g) ± SD
As_c_	0.21	0.24	0.24	0.23 ± 0.02
As_t_	1.32	1.21	1.27	1.26 ± 0.06
Ah_c_	0.31	0.35	0.43	0.36 ± 0.06
Ah_t_	2.07	2.31	2.43	2.27 ± 0.18

As_c_: control samples of *Amaranthus *sp. seeds, As_t_: treated samples of *Amaranthus *sp. seeds, Ah_c_: control samples of *A. hybridus* seeds, and Ah_t_: treated samples of *A. hybridus* seeds.

**Table 5:** Selenium concentration of soil samples.

Soil samples	Concentration *µ*g/g			Mean concentration, (*µ*g/g) ± SD
BSA	0.43	0.40	0.47	0.43 ± 0.03
ASA	18.43	29.43	22.78	23.55 ± 5.54
AVH	0.88	0.93	1.35	1.05 ± 0.26

BSA: before selenium application, ASA: after selenium application, and AVH: after vegetable harvest.

**Table 6:** Cytotoxicity evaluation of the water extracts against the human MDA-MB-231 cell line.

Vegetable leaves extract	Concentration					
	100 *µ*g/mL			<100 *µ*g/mL		
	A	B	C	A	B	C
SV	+	+	+	−	−	+
SS	+	+	+	−	−	−
IB	−	−	−	−	−	−
AH	−	−	−	−	−	−
AS	−	−	−	−	−	−
CM	−	−	−	−	−	−
VU	−	−	−	−	−	−

SV: *Solanum villosum*, SS: *Solanum scabrum*, IB: *Ipomoea batatas*, AH: *Amaranthus hybridus*, AS: *Amaranthus *sp., CM: *Cucurbita maxima*, VU: *Vigna unguiculata*, A: early harvest Se enriched samples, B: late harvest Se enriched samples, C: control samples, “+”: cytotoxicity observed, and “−”: no cytotoxicity observed.
